# The algorithm used for the calculation of gas exchange affects the estimation of O_2_ uptake kinetics at the onset of moderate‐intensity exercise

**DOI:** 10.1113/EP091146

**Published:** 2023-11-20

**Authors:** Maria Pia Francescato, Valentina Cettolo

**Affiliations:** ^1^ Department of Medicine University of Udine Udine Italy

**Keywords:** asymptotic standard error, fitting window, O_2_ deficit, phase 1‐phase 2 transition

## Abstract

At the start of a moderate‐intensity square‐wave exercise, after a short delay, breath‐by‐breath O_2_ uptake at the mouth is approximated to a mono‐exponential function, whose time constant is considered matched to that of the O_2_ uptake of the working muscles. We compared the kinetic parameters obtained from the breath‐by‐breath gas exchange data yielded by the ‘Independent‐breath’ algorithm (IND), which accounts for the changes in lung gas stores, with those obtained with the classical ‘Expiration‐only’ algorithm (EXP). The two algorithms were applied on the same flow and gas fraction traces acquired on 10 healthy volunteers, performing 10 times the same moderate‐intensity exercise transition. Repeated O_2_ uptake responses were stacked together and the kinetic parameters of a mono‐exponential function were estimated by non‐linear regression, removing the data pertaining to 1‐s progressively longer initial periods (Δ*T*_r_). Independently of Δ*T*_r_, the mean response time (time constant + time delay) obtained for the IND data was faster compared to the EXP data (∼43 s vs. ∼47 s, *P* < 0.001), essentially because of shorter time delays. Between Δ*T*_r_ = 16 s and Δ*T*_r_ = 29s, the time constants of the IND data decreased (30.7 s vs. 28.0 s, *P* < 0.05; drop = 10%), but less than those of the EXP data (32.2 s vs. 26.2 s, *P* < 0.001; drop = 23%); with the same Δ*T*_r_, the time constants of the two algorithms’ data were not different (*P* > 0.07). The different decrease in the time constant, together with the different mean response time, suggests that the data yielded by the two algorithms provide a different picture of the phenomena occurring at the beginning of the exercise.

## Abstract (highlights)

- **What is the central question of this study?**
  Is the kinetics of breath‐by‐breath gas exchange at the start of a square‐wave moderate intensity exercise faster when accounting for the changes in lung gas stores?
- **What is the main finding and its importance?**
  Accounting for the changes in lung gas stores, the time constant of the ${\dot{V}}_{O_{2}}$ kinetics changed less when the data pertaining to the initial period were progressively removed; time delays and mean response times were faster. Consequently, using different gas exchange calculation algorithms, the physiological phenomena occurring at the beginning of moderate‐intensity exercise are characterized differently.

## 1 INTRODUCTION

At the start of a moderate‐intensity square‐wave exercise, breath‐by‐breath O_2_ uptake (${\dot{V}}_{O_{2}}$) as assessed at the mouth lags behind the requested mechanical power for a few minutes (Ferretti et al., 2022). Two main phases are deemed to occur throughout this transient period. The first phase (Φ_1_) is believed to represent the cardiodynamic adjustment to the exercise, while the second one (Φ_2_, called primary phase) is assumed to be the image of the O_2_ uptake at the muscle level, and to be related to endurance performance (Bowen et al., 2019; Rossiter, 2011). Phase 2 is investigated by approximating the oxygen uptake at the mouth to a mono‐exponential function and the time constant (τ) of its kinetics, obtained by the non‐linear regression fitting procedure, is mostly the only evaluated parameter. However, the estimated τ might be affected by the amount of information for Φ_1_ included or excluded in or from the fitting window. Theoretically, if the mono‐exponential function adequately fits the time course of the original data, stable τ values should be reached after an initial decrease, as soon as the information pertaining to Φ_1_ is completely excluded (Rossiter et al., 1999). However, no stable τ values were found either in clean simulated ${\dot{V}}_{O_{2}}$ data (Benson et al., 2017) or in experimental data showing a low signal‐to‐noise ratio (Francescato & Cettolo, 2021).

The time course of oxygen consumption at the muscle level is believed to be better approximated by the gas exchange assessed at the mouth if the changes in lung gas stores are accounted for, as occurs with the ${\dot{V}}_{O_{2}}$ values yielded by the ‘Independent‐breath’ algorithm (Cettolo & Francescato, 2018a). This algorithm is based on the alternative view of the respiratory cycle of Grønlund (1984), and identifies the start and end points of the cycle on the basis of equal ratios between the end–tidal fractions of exchanged and not exchanged respiratory gases (Cettolo & Francescato, 2015). Conversely, the most commonly used algorithm is likely the ‘Expiration‐only’ one (Golja et al., 2018), which calculates gas exchange from information collected only during expiration, estimating the inspiratory volume through the Haldane transformation (Roecker et al., 2005; Ward, 2018) and identifying the start and end points of the respiratory cycle from the changes in the flow direction.

Changes in lung gas stores are expected mainly at the start of a square‐wave exercise (Wüst et al., 2008), and thus the use of different calculation algorithms might affect the temporal behaviour of gas exchange during this transient period. As a matter of fact, by comparing the kinetic parameters obtained for the ${\dot{V}}_{O_{2}}$ yielded by the ‘Independent‐breath’ algorithm with those provided by more commonly available algorithms, a faster kinetics was found when all the data pertaining to the transient period were included in the fitting window (Francescato & Cettolo, 2019). In that experimentation, however, the signal‐to‐noise ratio was poor because only one repetition of the square‐wave exercise was performed by each volunteer. It can be hypothesized that, by improving the signal‐to‐noise ratio, a faster kinetics will be found for the data yielded by the ‘Independent‐breath’ algorithm compared to those of the ‘Expiration‐only’ algorithm, even excluding from the fitting window the data pertaining to the initial time period (e.g. the data included in the first 20–25 s).

The aim of the present work was to test the above hypothesis by pooling together the breath‐by‐breath gas exchange data obtained during 10 repeated transitions performed by each volunteer, in order to increase the signal‐to‐noise ratio. For both algorithms, the parameters describing the timing of the kinetics were estimated several times by changing the length of the period excluded from the fitting window, in order to evaluate the effects of the inclusion/exclusion of interfering information, likely pertaining to Φ_1_.

## 2 METHODS

### 2.1 Experimental protocol

Ten healthy and moderately active adults (5 males and 5 females) volunteered to be subjects. Their mean (± SD) age, stature and body mass were 24.6 ± 3.6 years, 1.73 ± 0.09 m, and 73.5 ± 15.1 kg, respectively. The experimental protocol, design and methods conformed to the standards set by the *Declaration of Helsinki*, except for registration in a database, and were approved by the Institutional Review Board of the Department of Medicine of the University of Udine (Italy) (no. 07/2020_IRB issued on 5 March 2020). After having been thoroughly informed about the nature, purpose and possible risks of the investigation, written informed consent was obtained by all the volunteers prior to their participation.

All the volunteers were required to have completed the Covid‐19 vaccination protocol at least 15 days before the first experimental session and no one reported long‐term Covid‐19 symptoms. In addition, the last experimental session of one volunteer and the first one of the next volunteer were scheduled at least 1 week apart.

Each volunteer was submitted to respiratory gas collection at the mouth while pedalling as close as possible to 60 rpm on an ergometer (Corival; Lode B.V., Groningen, the Netherlands) during: (a) 5 min pedalling at 10 W (10W‐P), (b) the first bout of 6 min pedalling at ∼1 W kg^−1^ body mass (moderate intensity pedalling; MI‐P), (c) 10 min pedalling at 10 W, (d) the second bout of 6 min pedalling at ∼1 W kg^−1^ body mass, and (e) 8 min pedalling at 10 W. Overall duration of the experimental session was thus of 35 min, during which the volunteer repeated twice the same square‐wave exercise transition, each preceded by at least 5 min pedalling at 10 W. The experimental session was repeated 5 times by each volunteer, at least 1 day apart, thus allowing a total of 10 repetitions for each of them to be obtained. At the start of the first experimental session, each participant chose the cycling position that met his/her comfort requirements and the same position was subsequently used in all the following sessions.

Mechanical power, pedalling frequency, heart rate (HR), and flow ($\dot{V}$ ), as well as O_2_ and CO_2_ fractions ($F_{O_{2}}$ and $F_{CO_{2}}$, respectively) in inspired and expired air at the mouth were continuously acquired throughout the trial (Metalyzer 3B, Cortex GmbH, Liepzig, Germany). The metabolic unit automatically controlled the timings of the protocol, in particular the transitions between the 10W‐P and the MI‐P conditions. Before each experimental session, the analysers were calibrated following the procedures indicated by the manufacturer. Mean actual mechanical power during the experimental sessions was 71.5 ± 13.1 W.

### 2.2 Gas exchange calculations

Breath‐by‐breath oxygen consumption (${\dot{V}}_{O_{2}}$) was calculated by means of the ‘Independent‐breath’ algorithm (IND) according to the following equation:

(1) $${\dot{V}}_{O_{2}}^{\mathrm{IND}}=-\frac{\int_{t_{1}}^{t_{2}}\dot{V}\times F_{O_{2}}\;dt-\frac{F_{O_{2}}(t_{1})}{F_{N_{2}}(t_{1})}\times \int_{t_{1}}^{t_{2}}\dot{V}\times F_{N_{2}}\;dt}{t_{2}-t_{1}}.$$

where $F_{N_{2}}$ is the instantaneous nitrogen fraction (calculated as 1 – $F_{O_{2}}$ – $F_{CO_{2}}$); *t*_1_ and *t*_2_ correspond to the start and the end time points of the respiratory cycle, respectively, both identified on the trace of the $F_{O_{2}}$/$F_{N_{2}}$ ratio (Cettolo & Francescato, 2018a).

Using the same originally acquired flow and gas fraction traces, ${\dot{V}}_{O_{2}}$ was calculated also by means of the ‘Expiration‐only’ algorithm (EXP) according to the following equation:

(2) $${\dot{V}}_{O_{2}}^{\mathrm{EXP}}=-\frac{\int_{t_{e}}^{t_{i}}\dot{V}\times F_{O_{2}}\;dt-\frac{F_{IO_{2}}}{F_{IN_{2}}}\times \int_{t_{e}}^{t_{i}}\dot{V}\times F_{N_{2}}\;dt}{t_{i}-t_{i-1}}$$

where times *t*_i_ and *t*_e_ correspond to the time points where flow changes direction and the inspiration and expiration start, respectively; and $F_{IO_{2}}$ and $F_{IN_{2}}$ correspond to the inspired ambient oxygen and nitrogen fractions and were set to 20.93% and 79.02%, respectively.

The software calculated automatically also breath‐by‐breath carbon dioxide exhalation (namely, ${\dot{V}}_{CO_{2}}^{\mathrm{IND}}$ and ${\dot{V}}_{CO_{2}}^{\mathrm{EXP}}$) introducing the appropriate changes into the above equations, as well as the corresponding respiratory exchange ratios (i.e., RER^IND^ and RER^EXP^).

Both algorithms and details of the computations were described in Francescato and Cettolo (2019). As a result, three time series (namely, ${\dot{V}}_{O_{2}}$, ${\dot{V}}_{CO_{2}}$ and RER) were obtained for the IND and EXP algorithms, and thus two triplets were obtained for each experimental session for each volunteer, expressed in STPD conditions. No data were discarded (or filtered) on any of the time series before the subsequent analyses.

### 2.3 Statistics on the steady states of the individual time series

All the data were analysed with the R environment (R Core Team, 2020).

**FIGURE 1:**
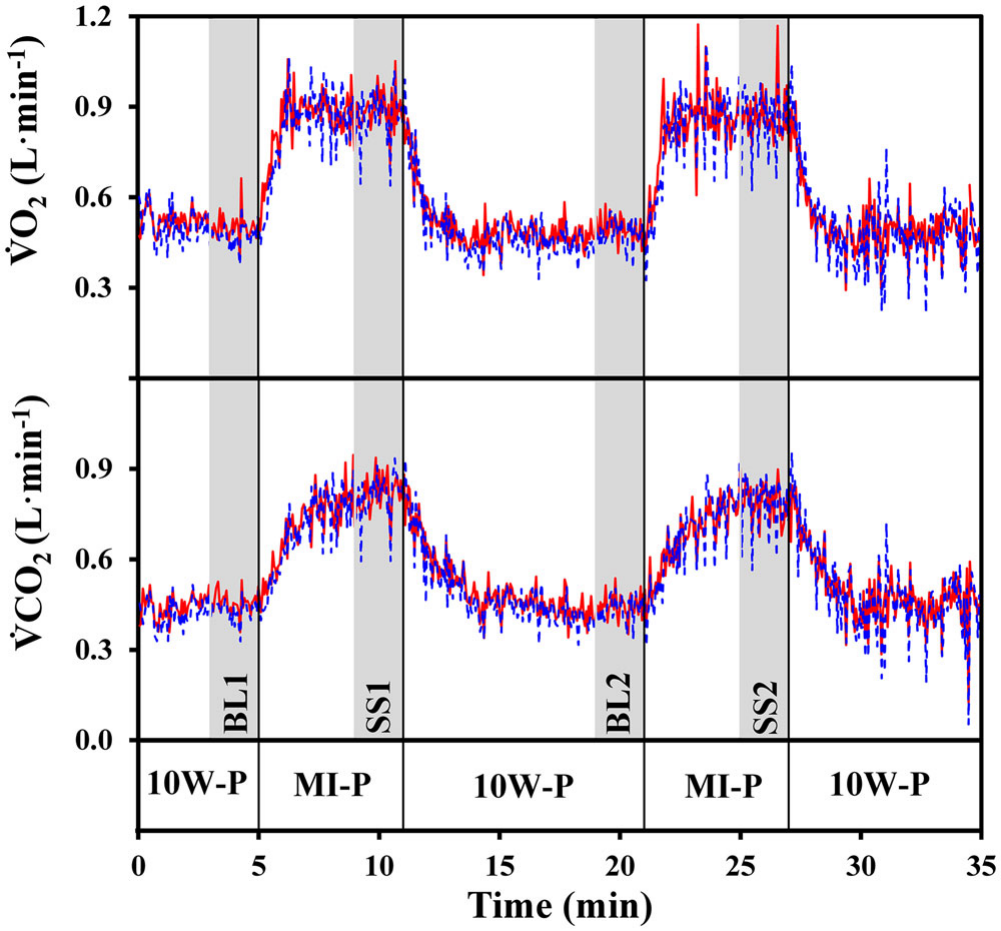
Example of gas exchange data obtained from one volunteer during an experimental session. The experimental protocol included: (a) an initial 5‐min pedalling period at 10 W (10W‐P), (b) two bouts of moderate intensity exercise, lasting 6 min each (1 W/kg, i.e., 55 W in this volunteer; MI‐P), separated by 10 min of pedalling at 10 W, and (c) a final recovery period lasting 8 min while pedalling at 10 W. Pedalling frequency was kept as close as possible to 60 rpm throughout. ${\dot{V}}_{O_{2}}$ (upper panel) and ${\dot{V}}_{CO_{2}}$ (lower panel) data obtained applying the IND (continuous red line) or the EXP (dashed blue line) algorithms are illustrated. Vertical lines correspond to the start and end times of the two bouts of moderate‐intensity pedalling periods. Shaded areas correspond to the time periods over which the data were averaged, namely, two periods during 10W‐P (BL1 and BL2) and two periods during MI‐P (SS1 and SS2).

For all the time series (namely, ${\dot{V}}_{O_{2}}^{\mathrm{IND}}$, ${\dot{V}}_{O_{2}}^{\mathrm{EXP}}$, ${\dot{V}}_{CO_{2}}^{\mathrm{IND}}$, ${\dot{V}}_{CO_{2}}^{\mathrm{EXP}}$, RER^IND^ and RER^EXP^), mean values, together with the corresponding standard deviations (SD), were calculated over the following four time periods: (a) baselines BL1 and BL2, namely the last 2 min just preceding each of the two transitions between 10W‐P and MI‐P, and (b) steady‐states SS1 and SS2, that is, the last 2 min of each of the two MI‐P bouts (Figure 1).

Significant differences for the means and the SDs were assessed applying an analysis of variance for repeated measures (2 × 2 × 5 ANOVA), with the following effects: between the values obtained during BL and SS (Load effect), between the two repetitions within the same experimental session (Repetition effect), and among the five experimental sessions (Session effect), as inter‐subject effects. In addition, the possible significant differences between the data yielded by the IND and the EXP algorithms were assessed (Algorithm effect). Post hoc pairwise comparison was used to detect significant differences among the various conditions.

### 2.4 Data treatment and statistics on the assembled time series

For each subject and each experimental session, all the obtained ${\dot{V}}_{O_{2}}$, ${\dot{V}}_{CO_{2}}$ and RER time series were split at *t* = 16 min, and the times were shifted setting the start of the two MI‐P bouts at *t* = 0. Subsequently, for each volunteer, the corresponding 10 repeated time series were assembled together using the stacking procedure (Francescato et al., 2014b).

Mean values and corresponding standard deviations were calculated over the same time periods described above, namely, for baseline (the last 2 min of 10W‐P just preceding *t* = 0 s; *A*_b_) and for steady‐state (the last 2 min of MI‐P just preceding *t* = 360 s; *A*_s_). These data allowed calculation of the ‘functional gain’ as the ratio between the net O_2_ uptake (i.e., *A*_s_ − *A*_b_) and the net mechanical power above baseline. In addition, over the same time periods, slopes of ${\dot{V}}_{O_{2}}$ against time were calculated by linear regression and their statistical significance was evaluated to assess the attainment of a steady condition.

The kinetic parameters of ${\dot{V}}_{O_{2}}$ and ${\dot{V}}_{CO_{2}}$ corresponding to the square‐wave exercise transition were obtained running the non‐linear regression procedure, using the R routine *nls.lm* (included in the *minpack.lm* library and minimizing the residual sum of squares by the Levenberg–Marquardt algorithm) without cleaning any outlier. The following mono‐exponential model was used:

(3) $$Y\left(t\right)=\left\{\begin{array}{c} \;A_{b}\;\;\;\;\;\;\;\;\;\;\;\;\;\;\;\;\;\;\;\;\;\;\;\;\;\;\;\;\;\;\;\;\;\;\;\;\;\;\;\;\;\;\;\;\;t<T_{d}\;\\ A_{b}+\Delta A\left(1-e^{-\;\frac{t-T_{d}}{\tau }}\right)\;\;\;\;\;\;\;\;\;t\geq T_{d} \end{array}\right.$$

The initial values to run the iterative non‐linear regression procedure were set to 25 s and 0 s for the time constant (τ) and the time delay (*T*_d_), respectively. Baseline signal was set to *A*_b_ (as calculated above), while the initial value for the signal change (Δ*A*) was set to *A*_s_ − *A*_b_, that is, the difference between the two signal intensities calculated above. The fitting procedure yielded the estimated values for τ, *T*_d_ and Δ*A*, and their asymptotic standard errors (ASE_τ_, ASE_Τd_ and ASE_Δ_ *_A_*, respectively), which allow evaluation of the specific confidence intervals of each estimated parameter (Francescato et al., 2014a, 2014b). In addition, the mean response time (MRT) of the overall kinetics was calculated for each data set as follows:

(4) $$\mathrm{MRT}=T_{d}+\tau .$$

The fitting procedure was run 51 times (always applying Equation 3), removing each time 1 s progressively longer time period (Δ*T*_r_) from the fitting window, starting from *t* = 0 s (i.e., Δ*T*_r_ ∈ [0 s, 50 s]). At the end of this procedure, 51 sets of estimated parameters (one for each Δ*T*_r_) were obtained for each volunteer and for the ${\dot{V}}_{O_{2}}$ and ${\dot{V}}_{CO_{2}}$ time series obtained for the two algorithms.

Student's two‐tailed *t*‐test for paired data was applied for each of the different Δ*T*_r_, to detect the statistically significant differences between the kinetic parameters obtained for the gas exchange time series provided by the two algorithms for each volunteer (*n* = 10). When a statistically significant difference was detected, its magnitude was evaluated by calculating the absolute value of the Cohen's *d*_Z_ effect size, where the Z alludes to the fact that the unit of analysis is the difference between the paired data, and the following absolute effect size classification was considered: ‘trivial’ < 0.2 < ‘small’ < 0.5 < ‘medium’ < 0.8 < ‘large’ < 1.3 < ‘very large’ (Lakens, 2013; Riemann & Lininger, 2018; Sullivan & Feinn, 2012).

All the statistically significant differences observed applying the parametric *t*‐test were confirmed by the Wilcoxon non‐parametric statistical test.

Summarized values are reported as means ± SD.

## 3 RESULTS

### 3.1 The experimental sessions

For all the investigated time series (calculated either by the IND and the EXP algorithms), no significant difference was observed among the mean values, or the corresponding standard deviations, when comparing the five different experimental sessions of each volunteer (repeated measures ANOVA, Session effect, *F* < 1.7, *P* > 0.17). Similarly, no significant difference was detected when comparing the two repetitions of the same experimental session (repeated measures ANOVA, Repetition effect, *F* < 3.6, *P* > 0.09). The mean values obtained for the moderate intensity pedalling condition, as well as their corresponding standard deviations, were significantly higher than those obtained for the 10W‐P condition (during SS vs. BL; repeated measures ANOVA, Load effect, *F* > 16.7, *P* < 0.003), with the exception of the standard deviations for RER (*F* = 1.2, *P* = 0.31).

The mean values obtained for the IND algorithm were not significantly different from those obtained for the EXP algorithm (repeated measures ANOVA, Algorithm effect, *F* < 2.2, *P* > 0.17). The standard deviations calculated for ${\dot{V}}_{O_{2}}^{\mathrm{IND}}$ were significantly lower than those obtained for ${\dot{V}}_{O_{2}}^{\mathrm{EXP}}$ (repeated measures ANOVA, Algorithm effect, *F* = 20.5, *P* = 0.001). No significant difference was detected for the ${\dot{V}}_{CO_{2}}$ values obtained with the two algorithms (*F* = 4.8, *P* = 0.06), whereas the RER^EXP^ showed significantly lower standard deviations compared to RER^IND^ (*F* = 476.3, *P* < 0.001).

Since no Repetition or Session effects were detected, the 10 time series of ${\dot{V}}_{O_{2}}$, ${\dot{V}}_{CO_{2}}$ and RER as obtained by each of the two algorithms were assembled together, thus yielding six stacked time series for each volunteer. Overall mean values and standard deviations were then calculated on the stacked time series and are summarized in Table 1, together with the corresponding ‘functional gains’. No significant differences were detected between the corresponding average values (*t* < 1.6, *P* > 0.14). The standard deviations were significantly smaller for the ${\dot{V}}_{O_{2}}^{\mathrm{IND}}$ data compared to the ${\dot{V}}_{O_{2}}^{\mathrm{EXP}}$ data (*t* > 3.5, *P* < 0.01, *d*_Z_ > 1.12), independent of the exercise intensity, whereas smaller standard deviations were observed for ${\dot{V}}_{CO_{2}}^{\mathrm{IND}}$ only during MI‐P (*t* = 3.0, *P* = 0.02, *d*_Z_ = 0.94). Significantly smaller standard deviations were obtained for RER^EXP^ compared to RER^IND^ (*t* > 9.0, *P* < 0.001, *d*_Z_ > 2.87).

**TABLE 1:** Mean values and means of the corresponding standard deviations (SD) of the stacked oxygen uptake (${\dot{V}}_{O_{2}}$), carbon dioxide exhalation (${\dot{V}}_{CO_{2}}$) and respiratory exchange ratio (RER) data yielded by the EXP and IND algorithms, for baseline and steady state time periods.

	IND		EXP	
	Mean	SD	Mean	SD
Baseline				
${\dot{V}}_{O_{2}}$ (l min^−1^)	0.572 (0.076)	0.082** (0.017)	0.569 (0.079)	0.099 (0.025)
${\dot{V}}_{CO_{2}}$ (l min^−1^)	0.491 (0.062)	0.085 (0.023)	0.488 (0.063)	0.090 (0.023)
RER	0.858 (0.029)	0.081 (0.016)	0.860 (0.027)	0.055*** (0.016)
Steady state				
${\dot{V}}_{O_{2}}$ (l min^−1^)	1.147 (0.196)	0.107*** (0.032)	1.156 (0.176)	0.148 (0.050)
${\dot{V}}_{CO_{2}}$ (l min^−1^)	1.016 (0.159)	0.114* (0.035)	1.028 (0.142)	0.137 (0.047)
RER	0.890 (0.039)	0.074 (0.013)	0.892 (0.037)	0.043*** (0.015)
Functional gain (ml min^−1^ W^−1^)	9.3 (0.5)		9.6 (0.5)	

*Note*: The table summarizes also the mean values of functional gain, calculated on the basis of the individual actual mechanical power. *n* = 10 (number of volunteers). Data in brackets are the standard deviations of the corresponding mean. Statistically significant different values by comparing the IND and EXP algorithms (two‐tailed paired *t*‐test, *P < 0.05; **P < 0.01; ***P < 0.005). The corresponding *d*_Z_ was >0.94 for the standard deviations, and >2.86 for the RER values.

Attainment of a steady state condition was confirmed for all the stacked ${\dot{V}}_{O_{2}}$ time series, their slopes being never significantly different from zero (*t* < 2.0, *P* > 0.05 in all cases).

### 3.2 The time period removed from the fitting window: effects on the ${\dot{V}}_{O_{2}}$ kinetic parameters

**FIGURE 2:**
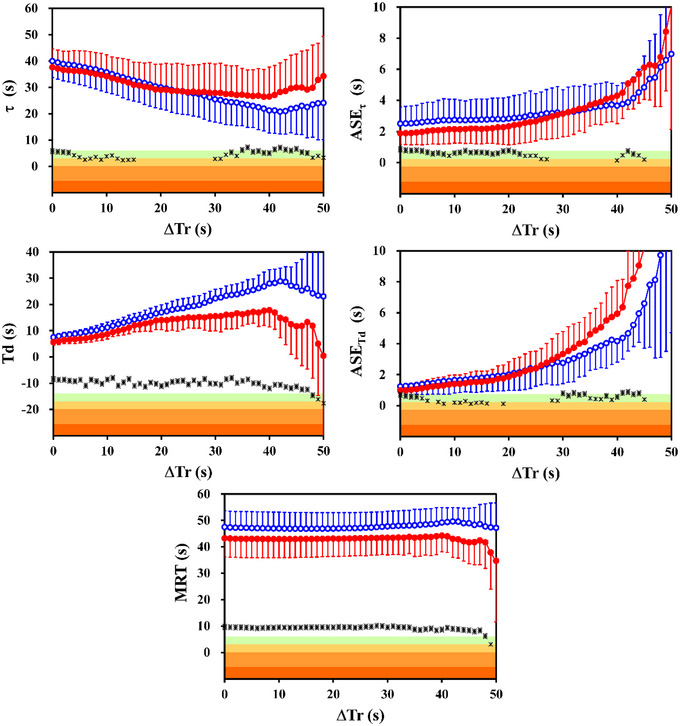
Mean time constants (τ; upper left panel) and mean time delays (*T*_d_; middle left panel) of oxygen uptake kinetics are illustrated as a function of the time period removed from the fitting window (Δ*T*_r_), as well as the corresponding mean asymptotic standard errors, ASE_τ_ (upper right panel) and ASE_Td_ (middle right panel). The corresponding mean response times (MRT) are illustrated in the lower panel. Results obtained for the data yielded by the IND (filled red circles) and the EXP (open blue circles) algorithms are illustrated in all panels. Vertical bars are the standard deviations of the individual parameters obtained running the non‐linear regression procedure with the mono‐exponential model. The differences between the two algorithms with the same Δ*T*_r_ are illustrated as Cohen's *d*_Z_ effect sizes (right vertical axis, on a logarithmic scale only for graphical purposes), using different symbols according to the statistical significance (*n* = 10; two‐tailed paired t‐test; * *P* < 0.05; ** *P* < 0.01). The 5 areas of decreasing colour intensity from bottom to top highlight the magnitude of Cohen's *d*_Z_ according to the commonly used thresholds (namely, ‘trivial’ < 0.2 < ‘small’ < 0.5 < ‘medium’ < 0.8 < ‘large’ < 1.3 < ‘very large’ with white background).

The stacked ${\dot{V}}_{O_{2}}^{\mathrm{IND}}$ time series resulted in a faster kinetics compared to the ${\dot{V}}_{O_{2}}^{\mathrm{EXP}}$ time series (Figure 2, left panels). As a matter of fact, up to Δ*T*_r_ < 16 s significantly shorter τ values were obtained for the stacked ${\dot{V}}_{O_{2}}^{\mathrm{IND}}$ time series (*t* > 2.32, *P* < 0.046, *d*_Z_ > 0.73; the difference being >4.0%). It is interesting to be noted that, between Δ*T*_r_ = 16 s and Δ*T*_r_ = 29 s, the time constants obtained for ${\dot{V}}_{O_{2}}^{\mathrm{IND}}$ vs. ${\dot{V}}_{O_{2}}^{\mathrm{EXP}}$ were not significantly different (*t* < 2.05, *P* > 0.07). Finally, for Δ*T*_r_ ≥ 30 s, the mean τ obtained for the stacked ${\dot{V}}_{O_{2}}^{\mathrm{EXP}}$ time series became significantly shorter than that for ${\dot{V}}_{O_{2}}^{\mathrm{IND}}$ (*t* > 2.50, *P* < 0.034, *d*_Z_ > 0.79, the difference being >9.1%). Conversely, the mean *T*_d_ (Figure 2, middle left panel) and the MRT (Figure 2, lower panel) obtained for the ${\dot{V}}_{O_{2}}^{\mathrm{IND}}$ data were significantly shorter for all the Δ*T*_r_ values up to 50 s (*t* > 2.60, *P* < 0.03, *d*_Z_ > 0.82 for both parameters). The difference was greater than 17% and 8% for *T*_d_ and MRT, respectively.

The ${\dot{V}}_{O_{2}}^{\mathrm{IND}}$ data provided more stable kinetic parameters as a function of Δ*T*_r_. In particular, by comparing Δ*T*_r_ = 0 s with Δ*T*_r_ = 40 s, the mean time constants (Figure 2, upper left panel) decreased with increasing Δ*T*_r_, with a maximum drop of 35% for the stacked ${\dot{V}}_{O_{2}}^{\mathrm{IND}}$ time series (*t* = 6.86, *P* < 0.001, *d*_Z_ = 2.17) which reached 61% for the ${\dot{V}}_{O_{2}}^{\mathrm{EXP}}$ time series (*t* = 15.4, *P* < 0.001, *d*_Z_ = 4.86). Focusing on the Δ*T*_r_ interval between 16 s and 29 s, the fall was still less pronounced for the ${\dot{V}}_{O_{2}}^{\mathrm{IND}}$ data (*t* = 2.82, *P* < 0.05, *d*_Z_ = 0.89; drop of 10%) compared to the ${\dot{V}}_{O_{2}}^{\mathrm{EXP}}$ data (*t* = 11.18, *P* < 0.001, *d*_Z_ = 3.54; drop of 23%). For both algorithms, the time delays increased by more than 100% from Δ*T*_r_ = 0 s to Δ*T*_r_ = 40 s (*t* > 5.70, *P* < 0.001, *d*_Z_ > 1.80). The resulting MRT increased significantly for the ${\dot{V}}_{O_{2}}^{\mathrm{EXP}}$ time series (*t* = 2.83, *P* = 0.02, *d*_Z_ = 2.66), whereas it remained constant for the ${\dot{V}}_{O_{2}}^{\mathrm{IND}}$ time series (*t* = 1.55, *P* = 0.15).

The mean ASE_τ_  values (Figure 2, upper right panel) obtained for the stacked ${\dot{V}}_{O_{2}}^{\mathrm{IND}}$ time series were statistically lower than those for the ${\dot{V}}_{O_{2}}^{\mathrm{EXP}}$ time series up to Δ*T*_r_ = 27 s (*t* > 2.53, *P* < 0.05, *d*_Z_ > 0.79, the difference being >10%). The corresponding mean ASE_Td_ values (Figure 2, middle right panel) were statistically lower up to Δ*T*_r_ = 16 s (*t* > 2.36, *P* < 0.05, *d*_Z_ > 0.62, the difference being >13%). All the mean ASE values increased with increasing Δ*T*_r_.

### 3.3 The time period removed from the fitting window: effects on the ${\dot{V}}_{CO_{2}}$ kinetic parameters

**FIGURE 3:**
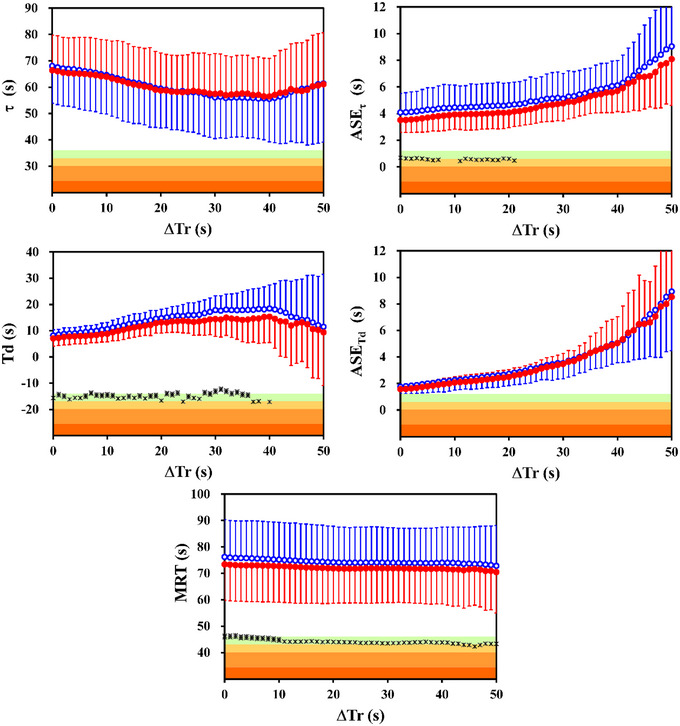
Mean time constants (τ; upper left panel) and mean time delays (*T*_d_; middle left panel) of carbon dioxide exhalation kinetics are illustrated as a function of the time period removed from the fitting window (Δ*T*_r_), as well as the corresponding mean asymptotic standard errors ASE_τ_ (upper right panel) and ASE_Td_ (middle right panel). The corresponding mean response times (MRT) are illustrated in the lower panel. Results obtained for the data yielded by the IND (filled red circles) and the EXP (open blue circles) algorithms are illustrated in all panels. Vertical bars are the standard deviations of the individual parameters obtained running the non‐linear regression procedure with the mono‐exponential model. The differences between the two algorithms with the same Δ*T*_r_ are illustrated as Cohen's *d*_Z_ effect sizes (right vertical axis, on a logarithmic scale only for graphical purposes), using different symbols according to the statistical significance (*n* = 10; two‐tailed paired *t*‐test; * *P* < 0.05; ** *P* < 0.01). The 5 areas of decreasing colour intensity from bottom to top highlight the magnitude of the Cohen's *d*_Z_ according to the commonly used thresholds (namely, ‘trivial’ < 0.2 < ‘small’ < 0.5 < ‘medium’ < 0.8 < ‘large’ < 1.3 < ‘very large’ with white background).

The time constants of the stacked ${\dot{V}}_{CO_{2}}^{\mathrm{IND}}$ time series were not significantly different from those of the ${\dot{V}}_{CO_{2}}^{\mathrm{EXP}}$ time series (Figure 3, upper left panel; *t* < 1.9, *P* > 0.09). Conversely, significantly shorter time delays were detected for ${\dot{V}}_{CO_{2}}^{\mathrm{IND}}$ up to Δ*T*_r_ = 40 s (*t* > 2.5, *P* < 0.04, *d*_Z_ > 0.70, the difference being greater than 8.5%). Even the MRT values (Figure 3, lower panel) obtained for the ${\dot{V}}_{CO_{2}}^{\mathrm{IND}}$ data were significantly shorter for Δ*T*_r_ up to 50 s (*t* > 2.3, *P* < 0.05, *d*_Z_ > 0.73), with a difference of about 3.0%.

For both algorithms, by comparing Δ*T*_r_ = 0 s with Δ*T*_r_ = 40 s, the mean time constants (Figure 2, upper left panel) decreased with increasing Δ*T*_r_, with a drop of ∼18% (*t* > 4.34, *P* < 0.002, *d*_Z_ > 1.38), the time delays increased by ∼76% (*t* > 3.21, *P* < 0.02, *d*_Z_ > 1.02), while the resulting MRT decreased (*t* > 2.75, *P* < 0.03, *d*_Z_ > 0.87, drop of ∼3%).

The mean ASE values (Figure 3, upper and middle right panels) obtained for the stacked ${\dot{V}}_{CO_{2}}^{\mathrm{IND}}$ time series were statistically smaller than those for the ${\dot{V}}_{CO_{2}}^{\mathrm{EXP}}$ time series only occasionally, mainly for the time constants and for Δ*T*_r_ up to 21 s. For both parameters, the mean ASE values increased with increasing Δ*T*_r_.

## 4 DISCUSSION

The main result of the present investigation was that the time parameters that characterize the oxygen uptake (or carbon dioxide exhalation) kinetics during moderate intensity exercise differed depending on the algorithm used to calculate the gas exchange data. In particular, our results suggest that the temporal behaviour of the ${\dot{V}}_{O_{2}}^{\mathrm{IND}}$ data, compared to that of ${\dot{V}}_{O_{2}}^{\mathrm{EXP}}$ data, resulted in a steadier time constant upon removing increasing time periods from the fitting window. Moreover, the ${\dot{V}}_{O_{2}}^{\mathrm{IND}}$ data showed a faster overall kinetics, as suggested by a shorter mean response time.

### 4.1 Comparison of the two algorithms under steady conditions

In the present experimentation the IND and EXP algorithms provided similar mean gas exchange values despite the EXP algorithm (Equation 2) was neglecting the dead space of the breathing apparatus. It should be noted, however, that the latter adjustment as proposed by Beaver et al. (1973) is under debate, considering that it should account even for breathing frequency and/or tidal volume (Ward, 2018). The mean oxygen uptake values are in agreement with the literature. Indeed, applying Equation 4 of Formenti et al. (2015), the estimated ${\dot{V}}_{O_{2}}$ will amount to 1.15 litres min^−1^, which is practically equal to the values reported in Table 1 (∼1.16 litres min^−1^). Moreover, the ‘functional gain’ of the responses (∼9.5 ml min^−1^ W^−1^) is not far from 9.47 ± 0.85 and ∼10.0 ml min^−1^ W^−1^, as reported by Benson et al. (2017) and Rossiter (2011), respectively. Although we are aware that the RER values are affected by the duration of fasting before the exercise (Liu & Chen, 2022), no attention was paid to performing all the experimental sessions at the same day time, since RER was not a main outcome. The obtained RER values, however, were in line with the values reported in the literature for similar exercise conditions (Baldassarre et al., 2022; Liu & Chen, 2022; Miyamoto et al., 2022).

As already observed under several different exercise conditions (Cettolo & Francescato, 2018a; Francescato & Cettolo, 2019; Koschate et al., 2019), the standard deviations of the ${\dot{V}}_{O_{2}}^{\mathrm{IND}}$ values during steady conditions were significantly lower than those of ${\dot{V}}_{O_{2}}^{\mathrm{EXP}}$. Indeed, the IND algorithm accounts for the changes of the pulmonary O_2_ stores, which typically occur for the anomalous breaths (Francescato et al., 2019), thus reducing the fluctuations around the mean of the oxygen consumption as measured at the mouth.

Similar standard deviations were observed between the ${\dot{V}}_{CO_{2}}^{\mathrm{IND}}$ and ${\dot{V}}_{CO_{2}}^{\mathrm{EXP}}$ values, in particular at rest. It is to be noted, however, that the alveolar CO_2_ stores are dynamically balanced with the amount of HCO_3_^−^ dissolved in the blood (Cettolo & Francescato, 2018b); accordingly, when an anomalous breath occurs, the CO_2_ lung stores might remain quite constant, making negligible the correction for their changes.

### 4.2 The ${\dot{V}}_{O_{2}}$ ON transient phase

Theoretically, as soon as all the information pertaining to Φ_1_ is excluded, the behaviour of the time constant of ${\dot{V}}_{O_{2}}$ as a function of Δ*T*_r_ should reach a stable value (Rossiter et al., 1999). This behaviour was not followed by the mean τ value of ${\dot{V}}_{O_{2}}^{\mathrm{EXP}}$ of the present investigation, which continued decreasing up to Δ*T*_r_ ≈ 40 s. Conversely, a rather stable mean τ value for ${\dot{V}}_{O_{2}}^{\mathrm{IND}}$ was observed for Δ*T*_r_ > 16 s suggesting that, for these fitting windows, information pertaining to phase 1 was likely excluded. Since the flow and gas traces, as collected at the mouth, were the same for the calculations with both algorithms, it could be expected that the cardiodynamic adjustment to the exercise (e.g. the increase in cardiac output) would be excluded for the same Δ*T*_r_, which was not the case. Consequently, the observed discrepancy can be explained only by the fact that the two algorithms account (or not) for the changes in lung gas stores in their calculations.

The model commonly used to estimate the kinetic parameters by non‐linear regression includes also the time delay, which mathematically is the back projection of the fitted data to the baseline. This parameter is correlated to the Φ_1_ to Φ_2_ transition time, although it does not correspond to the latter (Rossiter, 2011). However, it should be noted that the sum of *T*_d_ and τ reflects the MRT of the overall O_2_ uptake kinetics during the transient and allows calculation of the O_2_ deficit (O_2def_) as follows (Whipp et al., 1982):

(5) $$O_{2\mathrm{def}}=\Delta A\times \left(T_{d}+\tau \right)=\Delta A\times \mathrm{MRT}.$$

Accordingly, there is a direct proportion between MRT and O_2_ deficit with equal Δ*A* (i.e. the net increase in O_2_ uptake at the asymptote). The O_2def_ includes the changes in the pulmonary O_2_ stores, which are theoretically compensated for by the IND algorithm. A faster MRT can thus be expected for the data of the IND algorithm compared to the EXP one, as was observed in the present work (43.3 ± 7.1 s vs. 47.5 ± 6.2 s).

The O_2_ deficit is a physiological variable that should be independent of the amount of information used by the non‐linear fitting procedure. Consequently, a quite constant MRT should be predicted irrespective of Δ*T*_r_, as was the case for both algorithms up to Δ*T*_r_ ≈ 40 s (Figure 2, lower panel), mainly because of complementary time constants and time delays.

For all the estimated parameters, the asymptotic standard error, namely the statistical descriptor that allows evaluation of the confidence intervals of the corresponding estimated value (Francescato et al., 2014a, 2014b), increased on average with increasing Δ*T*_r_, likely because fewer data points were included in the non‐linear regression. Smaller ASE values were obtained for ${\dot{V}}_{O_{2}}^{\mathrm{IND}}$ compared to ${\dot{V}}_{O_{2}}^{\mathrm{EXP}}$, in particular for the smaller Δ*T*_r_ (Figure 2, right panels). These results suggest that the IND algorithm provides less noisy gas exchange data also during the transient phases and/or the ${\dot{V}}_{O_{2}}^{\mathrm{IND}}$ data fit better the mono‐exponential model.

### 4.3 The ${\dot{V}}_{CO_{2}}$ ON transient phase

To the best of our knowledge, the kinetics of carbon dioxide exhalation at the start of a moderate intensity exercise has received much less attention in the literature compared to the kinetics of oxygen uptake, despite its providing insights into the inherent control features of ventilation and gas exchange (Ward et al., 2023; Whipp et al., 1982). It can be hypothesized that the cardiodynamic adjustment at the beginning of the exercise affects also the breath‐by‐breath ${\dot{V}}_{CO_{2}}$ values, involving changes in lung gas stores. Indeed, similar to the behaviour of the kinetic parameters of ${\dot{V}}_{O_{2}}$, in the present investigation, the time constant of ${\dot{V}}_{CO_{2}}$ decreased with increasing Δ*T*_r_, while the *T*_d_ increased (Figure 3, left panels). Nevertheless, no quite steady ${\dot{V}}_{CO_{2}}$ kinetic parameters were found by removing from the fitting window 1‐s longer initial periods, suggesting that the behaviour of ${\dot{V}}_{CO_{2}}$ at the start of the exercise departs from the mono‐exponential function much more than the kinetics of ${\dot{V}}_{O_{2}}$.

In addition, results of the present work suggest that the changes in lung CO_2_ stores are less pronounced compared to the changes in lung O_2_ stores (both accounted for by the IND algorithm). Indeed, these changes were not sufficient to affect the values of the ${\dot{V}}_{CO_{2}}$ time constant, and/or the corresponding ASE values, which were not significantly different between the two algorithms at stake for the majority of the explored Δ*T*_r_. The lung CO_2_ stores, however, changed to an extent detectable by the IND algorithm, and influenced slightly the start of the kinetics, resulting in a lower time delay and/or mean response time.

### 4.4 The commonly used procedure of analysis

**FIGURE 4:**
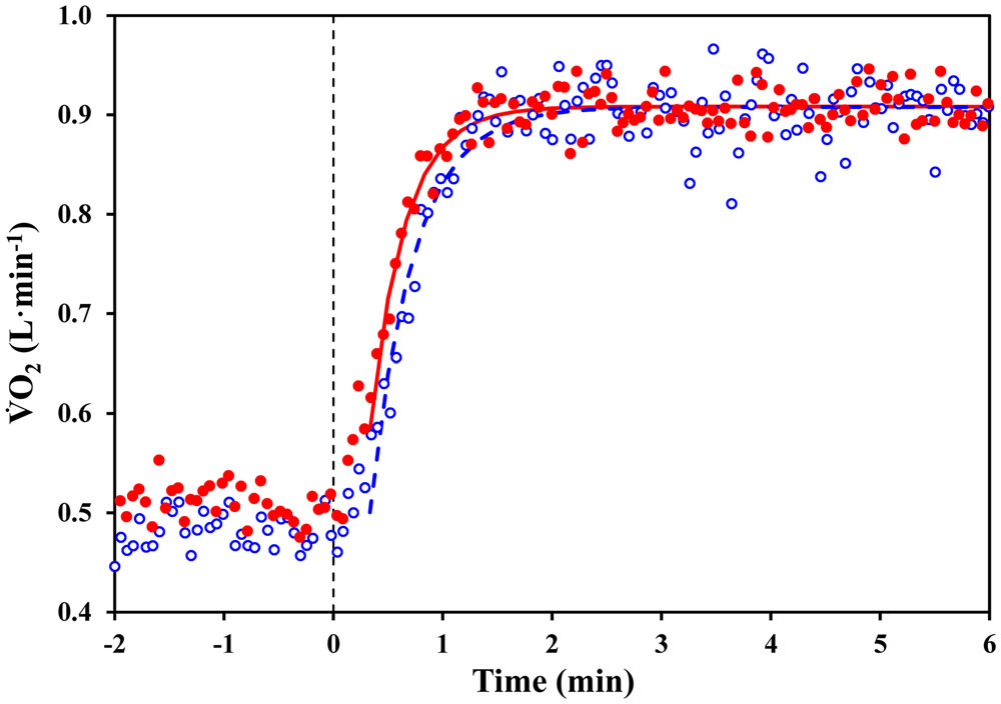
Stacked ${\dot{V}}_{O_{2}}^{\mathrm{IND}}$ (filled red circles; continuous line) and ${\dot{V}}_{O_{2}}^{\mathrm{EXP}}$ (open blue circles; dashed line) time series of the same subjects as in Figure 1. For graphical purposes, data of the 10 repetitions pertaining to the same algorithm were first stacked together, sorted in ascending time and then averaged both in time and in signal intensity over 10 consecutive data points (i.e. the number of repetitions). For both algorithms, the parameters estimated by non‐linear regression with Δ*T*_r_ = 20 s were used to draw the two lines showing the corresponding kinetics.

Focusing specifically on the arbitrary time period excluded by the vast majority of those estimating ${\dot{V}}_{O_{2}}$ kinetics (i.e., ∆*T*_r_ = 20 s, which does not necessarily correspond to the Φ_1_ to Φ_2_ transition time), no significant difference in the average time constant was detected between the ${\dot{V}}_{O_{2}}^{\mathrm{IND}}$ and ${\dot{V}}_{O_{2}}^{\mathrm{EXP}}$ data (29.0 ± 9.8 s vs. 30.0 ± 8.2 s, respectively). These averages might lead to the conclusion that there is no effective difference between using the IND or EXP algorithms. It cannot be excluded, however, that the similarity of the τ estimated for the data provided by the two algorithms with Δ*T*_r_ ≈ 20 s was observed by chance, or it is linked to the characteristics (among which age and training level) of the recruited subjects. Indeed, phase 1 in older volunteers can be as long as 35 s (Mezzani et al., 2010), and thus the data of the two algorithms might produce significantly different time constants at Δ*T*_r_ of 20 s. Despite the similar time constants, it is not possible to neglect the significantly shorter *T*_d_ for the IND algorithm (14.1 ± 3.6 s vs. 16.9 ± 2.9 s, for ${\dot{V}}_{O_{2}}^{\mathrm{IND}}$ and ${\dot{V}}_{O_{2}}^{\mathrm{EXP}}$, respectively), which is suggestive of a faster overall kinetics. Figure 4 illustrates the two stacked ${\dot{V}}_{O_{2}}$ time series of the same subject as for Figure 1, together with the regression lines corresponding to the two kinetics obtained for Δ*T*_r_ = 20 s. It clearly appears that the kinetics for the ${\dot{V}}_{O_{2}}^{\mathrm{IND}}$ data were faster compared to the ${\dot{V}}_{O_{2}}^{\mathrm{EXP}}$ data. Only for graphical reasons, in Figure 4 the data of the 10 repetitions were first stacked together, sorted in ascending time and then averaged both in time and in signal intensity over 10 consecutive data points (i.e. the number of repetitions). Finally, it should be noted that, for Δ*T*_r_ = 20 s, the ${\dot{V}}_{O_{2}}^{\mathrm{IND}}$ data resulted in smaller ASE values, suggesting that the corresponding estimated parameters are more precise (Figure 2 right panels).

In the present experimentation, volunteers repeated 10 times the same exercise transient. Nevertheless, when the number of repetitions was limited to four, as suggested by Benson et al. (2017), results were comparable to those obtained on 10 repeated bouts, as concerned all the investigated areas, that is, the mean values and corresponding standard deviations, the statistically significant differences between the two algorithms, and, finally, the behaviours according to the excluded time period (Δ*T*_r_). As expected, however, the average ASE values obtained for the data yielded by both algorithms were somewhat greater than those obtained for 10 repetitions, while the ${\dot{V}}_{O_{2}}^{\mathrm{IND}}$ data still resulted in lower ASE values compared to the ${\dot{V}}_{O_{2}}^{\mathrm{EXP}}$ data (data not shown).

### 4.5 Fraction of pulmonary O_2_ stores accounted for by the IND algorithm

As already stated above, the MRT of ${\dot{V}}_{O_{2}}$ at the start of a square‐wave exercise of moderate intensity allows calculation of the O_2_ deficit (Whipp et al., 1982). A smaller O_2_ deficit could be estimated for the ${\dot{V}}_{O_{2}}^{\mathrm{IND}}$ data compared to the ${\dot{V}}_{O_{2}}^{\mathrm{EXP}}$ data (i.e., ∼420 mL vs. ∼470 mL), because of the ∼10% difference between the MRT values obtained for the two algorithms. The difference of ∼50 mL in the O_2_ deficit might be associated to the changes in lung O_2_ stores, likely accounted for by the IND algorithm, but not by the EXP one. This volume might seem small and thus negligible, but this is not the case, since it represents about 56% of the overall changes in the pulmonary O_2_ stores (50 mL/90 mL).

Indeed, the overall changes in pulmonary O_2_ stores (∼90 mL) were estimated assuming that: (a) the splitting of the high‐energy phosphates is the mirror image of oxygen consumption at the muscle level (Ferretti et al., 2022; Rossiter et al., 1999), with a time constant of 25 s without any time delay (Barker et al., 2008; Francescato et al., 2013; Jones et al., 2008), and (b) the changes in the venous blood O_2_ stores can be estimated according to the arterio‐ to mixed venous blood oxygen difference at rest and at steady state (Francescato et al., 2003), using the values reported for an exercise similar to that of the present investigation (Fontolliet et al., 2021).

### 4.6 Strengths and limitations

Volunteers started abruptly the moderate intensity exercise bout after a 10‐W pedalling period, ensuring that the increase in O_2_ uptake was essentially due to the increase in mechanical power and not to the inertial effects of the ergometer flywheel at exercise onset (Whipp et al., 1982) or to the work needed to move the limbs (Francescato et al., 1995). Even possible interferences of anticipation were avoided (Miyamoto et al., 2022), since volunteers were not informed about the timings of the protocol, in particular of the changes in workload, that were automatically set by the software of the metabolic unit. This allowed us to circumvent the need to apply manual re‐alignments specific for each volunteer.

Exercise intensity was chosen according to volunteer's body mass, not relative to an individual physiological threshold during exercise (e.g. gas exchange threshold or lactate threshold). During a constant intensity exercise below the above thresholds, however, following the cardiodynamic phase and the primary phase, gas exchange remains quite constant and does not show a temporal drift (the so‐called slow component). The non‐statistically significant slopes found in the gas exchange data during steady‐state are suggestive of the absence of a slow component in all the volunteers. Moreover, since the same original flow and gas fraction traces were used to calculate gas exchange by means of the two algorithms, the slow component would have had a similar effect on whatever kinetic parameter (e.g. lengthening the time constant) obtained for both algorithms.

Each volunteer repeated 10 times the same moderate intensity pedalling bout and repeatable data were collected making us confident that our experimental data show an adequate signal‐to‐noise ratio for kinetic analyses. Moreover, the different repetitions were stacked, without discarding or filtering the originally acquired data, maintaining the validity and meaning of every single value in determining the kinetic parameters and the corresponding uncertainty (Francescato et al., 2014b, 2015, 2017).

The group of volunteers was rather uniform in age, allowing us to obtain more homogeneous results and to make inferences on the basis of literature data for volunteers of similar characteristics (i.e., the phosphocreatine breakdown time constant and the arterio‐ to mixed venous blood oxygen difference at rest and during exercise). Conversely, the narrow range of ages might be considered a limitation, since older subjects might lead to different results.

Finally, the comparison of gas exchange kinetic parameters for ${\dot{V}}_{O_{2}}$ data obtained with different calculation methods/algorithms has led to contrasting results (e.g., Aliverti et al., 2009; Beaver et al., 1981; Cautero et al., 2002), likely because of different experimental protocols (e.g. different baselines) and/or methods of analysis (e.g. fitting with a bi‐exponential model). Consequently, a systematic comparison with our results could only introduce further confusion.

It remains to be evaluated whether the two algorithms at stake might show a different sensitivity in detecting intervention‐induced changes in the gas exchange kinetic parameters (e.g. exercise training). It can be expected that the IND algorithm might detect differently the interventions that change the Φ_1_Φ_2_ transition time and/or that change the involvement of the lung gas stores.

### 4.7 Conclusions

Results of the present investigation suggest that, using a mono‐exponential model, the kinetic parameters of breath‐by‐breath oxygen uptake and carbon dioxide exhalation might differ depending on whether gas exchange is calculated accounting for the changes in lung gas stores or not. The IND and EXP algorithms resulted in comparable oxygen uptake time constants only for a portion of the investigated fitting windows (i.e., for Δ*T*_r_ between 16 s and 29 s), while the time constants of carbon dioxide exhalation were comparable at all the investigated Δ*T*_r_. Nevertheless, the IND algorithm yielded significantly faster mean response times for ${\dot{V}}_{O_{2}}$ over the whole range of investigated Δ*T*_r_, to which corresponded a smaller O_2_ deficit. Consequently, the physiological phenomena occurring at the start of a moderate intensity exercise might be interpreted differently according to the gas exchange calculation algorithm used.

## AUTHOR CONTRIBUTIONS

Maria Pia Francescato and Valentina Cettolo contributed in conception and design of the experiments; both performed the experiments, analysed the data and wrote the paper. All authors have read and approved the final version of this manuscript and agree to be accountable for all aspects of the work in ensuring that questions related to the accuracy or integrity of any part of the work are appropriately investigated and resolved. All persons designated as authors qualify for authorship, and all those who qualify for authorship are listed.

## CONFLICT OF INTEREST

The authors declare no conflicts of interest.

## Data Availability

Data supporting the findings of the present paper as well as the software used are available from the corresponding author upon reasonable request.

## References

[eph13457-bib-0001] Aliverti, A. , Kayser, B. , Cautero, M. , Dellaca, R. L. , di Prampero, P. E. , & Capelli, C. (2009). Pulmonary VO_2_ kinetics at the onset of exercise is faster when actual changes in alveolar O_2_ stores are considered. Respiratory Physiology & Neurobiology, 169, 78–82. 19715776 10.1016/j.resp.2009.08.012

[eph13457-bib-0002] Baldassarre, G. , Zuccarelli, L. , Manferdelli, G. , Manfredini, V. , Marzorati, M. , Pilotto, A. , Porcelli, S. , Rasica, L. , Šimunič, B. , Pišot, R. , Narici, M. , & Grassi, B. (2022). Decrease in work rate in order to keep a constant heart rate: Biomarker of exercise intolerance following a 10‐day bed rest. Journal of Applied Physiology, 132, 1569–1579. 35511721 10.1152/japplphysiol.00052.2022

[eph13457-bib-0003] Barker, A. R. , Welsman, J. R. , Fulford, J. , Welford, D. , & Armstrong, N. (2008). Muscle phosphocreatine kinetics in children and adults at the onset and offset of moderate‐intensity exercise. Journal of Applied Physiology, 105, 446–456. 18499782 10.1152/japplphysiol.00819.2007

[eph13457-bib-0004] Beaver, W. L. , Lamarra, N. , & Wasserman, K. (1981). Breath‐by‐breath measurement of true alveolar gas exchange. Journal of Applied Physiology: Respiratory, Environmental and Exercise Physiology, 51, 1662–1675. 6798003 10.1152/jappl.1981.51.6.1662

[eph13457-bib-0005] Beaver, W. L. , Wasserman, K. , & Whipp, B. J. (1973). On‐line computer analysis and breath‐by‐breath graphical display of exercise function tests. Journal of Applied Physiology, 34, 128–132. 4697371 10.1152/jappl.1973.34.1.128

[eph13457-bib-0006] Benson, A. , Bowen, T. , Ferguson, C. , Murgatroyd, S. , & Rossiter, H. B. (2017). Data collection, handling and fitting strategies to optimize accuracy and precision of oxygen uptake kinetics estimation from breath‐by‐breath measurements. Journal of Applied Physiology, 123, 227–242. 28450551 10.1152/japplphysiol.00988.2016

[eph13457-bib-0007] Bowen, T. S. , Benson, A. P. , & Rossiter, H. B. (2019). Chapter 10 ‐ The coupling of internal and external gas exchange during exercise. In J. A. Zoladz (Ed.), Muscle and Exercise Physiology. Academic Press (pp. 217–249).

[eph13457-bib-0008] Cautero, M. , Beltrami, A. P. , di Prampero, P. E. , & Capelli, C. (2002). Breath‐by‐breath alveolar oxygen transfer at the onset of step exercise in humans: Methodological implications. European Journal of Applied Physiology, 88, 203–213. 12458363 10.1007/s00421-002-0671-8

[eph13457-bib-0009] Cettolo, V. , & Francescato, M. P. (2015). Assessment of breath‐by‐breath alveolar gas exchange: An alternative view of the respiratory cycle. European Journal of Applied Physiology, 115, 1897–1904. 25893561 10.1007/s00421-015-3169-x

[eph13457-bib-0010] Cettolo, V. , & Francescato, M. P. (2018a). Assessing breath‐by‐breath alveolar gas exchange: Is the contiguity in time of breaths mandatory? European Journal of Applied Physiology, 118, 1119–1130. 29546638 10.1007/s00421-018-3842-y

[eph13457-bib-0011] Cettolo, V. , & Francescato, M. P. (2018b). Effects of abrupt changes in lung gas stores on the assessment of breath‐by‐breath gas exchange. Clinical Physiology and Functional Imaging, 38, 491–496. 28574212 10.1111/cpf.12444

[eph13457-bib-0012] Ferretti, G. , Fagoni, N. , Taboni, A. , Vinetti, G. , & di Prampero, P. E. (2022). A century of exercise physiology: Key concepts on coupling respiratory oxygen flow to muscle energy demand during exercise. European Journal of Applied Physiology, 122, 1317–1365. 35217911 10.1007/s00421-022-04901-x PMC9132876

[eph13457-bib-0013] Fontolliet, T. , Bringard, A. , Adami, A. , Fagoni, N. , Tam, E. , Taboni, A. , & Ferretti, G. (2021). Vagal blockade suppresses the phase I heart rate response but not the phase I cardiac output response at exercise onset in humans. European Journal of Applied Physiology, 121, 3173–3187. 34390402 10.1007/s00421-021-04769-3 PMC8505324

[eph13457-bib-0014] Formenti, F. , Minetti, A. E. , & Borrani, F. (2015). Pedaling rate is an important determinant of human oxygen uptake during exercise on the cycle ergometer. Physiological Reports, 3, e12500. 26371230 10.14814/phy2.12500 PMC4600374

[eph13457-bib-0015] Francescato, M. , Cettolo, V. , & Bellio, R. (2014a). Confidence intervals for the parameters estimated from simulated O_2_ uptake kinetics: Effects of different data treatments. Experimental Physiology, 99, 187–195. 24121286 10.1113/expphysiol.2013.076208

[eph13457-bib-0016] Francescato, M. P. , & Cettolo, V. (2019). The “independent breath” algorithm: Assessment of oxygen uptake during exercise. European Journal of Applied Physiology, 119, 495–508. 30515592 10.1007/s00421-018-4046-1

[eph13457-bib-0017] Francescato, M. P. , & Cettolo, V. (2021). Influence of the fitting window on the O_2_ uptake kinetics at the onset of moderate intensity exercise. Journal of Applied Physiology, 131, 1009–1019. 34292790 10.1152/japplphysiol.00154.2021

[eph13457-bib-0018] Francescato, M. P. , Cettolo, V. , & Bellio, R. (2014b). Assembling more O_2_ uptake responses: Is it possible to merely stack the repeated transitions? Respiratory Physiology & Neurobiology, 200, 46–49. 24927877 10.1016/j.resp.2014.06.004

[eph13457-bib-0019] Francescato, M. P. , Cettolo, V. , & Bellio, R. (2015). Interpreting the confidence intervals of model parameters of breath‐by‐breath pulmonary O_2_ uptake. Experimental Physiology, 100, 475. 25833110 10.1113/EP085043

[eph13457-bib-0020] Francescato, M. P. , Cettolo, V. , & Bellio, R. (2017). Interpreting the averaging methods to estimate oxygen uptake kinetics parameters. Journal of Applied Physiology, 123, 1018–1018. 29074572 10.1152/japplphysiol.00494.2017

[eph13457-bib-0021] Francescato, M. P. , Cettolo, V. , & Di Prampero, P. E. (2003). Relationships between mechanical power, O(2) consumption, O(2) deficit and high‐energy phosphates during calf exercise in humans. Pflugers Archiv: European Journal of Physiology, 445, 622–628. 12634935 10.1007/s00424-002-0992-9

[eph13457-bib-0022] Francescato, M. P. , Cettolo, V. , & di Prampero, P. E. (2013). Oxygen uptake kinetics at work onset: Role of cardiac output and of phosphocreatine breakdown. Respiratory Physiology & Neurobiology, 185, 287–295. 23043876 10.1016/j.resp.2012.09.015

[eph13457-bib-0023] Francescato, M. P. , Girardis, M. , & di Prampero, P. E. (1995). Oxygen cost of internal work during cycling. European Journal of Applied Physiology and Occupational Physiology, 72, 51–57. 8789570 10.1007/BF00964114

[eph13457-bib-0024] Francescato, M. P. , Thieschäfer, L. , Cettolo, V. , & Hoffmann, U. (2019). Comparison of different breath‐by‐breath gas exchange algorithms using a gas exchange simulation system. Respiratory Physiology & Neurobiology, 266, 171–178. 31009753 10.1016/j.resp.2019.04.009

[eph13457-bib-0025] Golja, P. , Cettolo, V. , & Francescato, M. P. (2018). Calculation algorithms for breath‐by‐breath alveolar gas exchange: The unknowns!. European Journal of Applied Physiology, 118, 1869–1876. 29938338 10.1007/s00421-018-3914-z

[eph13457-bib-0026] Grønlund, J. (1984). A new method for breath‐to‐breath determination of oxygen flux across the alveolar membrane. European Journal of Applied Physiology, 52, 167–172. 10.1007/BF00433387 6232137

[eph13457-bib-0027] Jones, A. M. , Wilkerson, D. P. , & Fulford, J. (2008). Muscle [phosphocreatine] dynamics following the onset of exercise in humans: The influence of baseline work‐rate. The Journal of Physiology, 586, 889–898. 18063663 10.1113/jphysiol.2007.142026 PMC2375627

[eph13457-bib-0028] Koschate, J. , Cettolo, V. , Hoffmann, U. , & Francescato, M. P. (2019). Breath‐by‐breath oxygen uptake during running: Effects of different calculation algorithms. Experimental Physiology, 104, 1829–1840. 31583757 10.1113/EP087916

[eph13457-bib-0029] Lakens, D. (2013). Calculating and reporting effect sizes to facilitate cumulative science: A practical primer for t‐tests and ANOVAs. Frontiers in Psychology, 4, 863. 24324449 10.3389/fpsyg.2013.00863 PMC3840331

[eph13457-bib-0030] Liu, M.‐Y. , & Chen, S.‐Q. (2022). Effects of low/medium‐intensity exercise on fat metabolism after a 6‐h fast. International Journal of Environmental Research and Public Health, 19, 15502. 36497577 10.3390/ijerph192315502 PMC9736603

[eph13457-bib-0031] Mezzani, A. , Grassi, B. , Giordano, A. , Corrà, U. , Colombo, S. , & Giannuzzi, P. (2010). Age‐related prolongation of phase I of VO_2_ on‐kinetics in healthy humans. American Journal of Physiology. Regulatory, Integrative and Comparative Physiology, 299, R968–R976. 20610830 10.1152/ajpregu.00739.2009

[eph13457-bib-0032] Miyamoto, T. , Sotobayashi, D. , Ito, G. , Kawai, E. , Nakahara, H. , Ueda, S. , Toyama, T. , Saku, K. , Nakanishi, Y. , & Kinoshita, H. (2022). Physiological role of anticipatory cardiorespiratory responses to exercise. Physiological Reports, 10, e15210. 35246949 10.14814/phy2.15210 PMC8897741

[eph13457-bib-0033] R Core Team . (2020). R: A Language and Environment for Statistical Computing. R Foundation for Statistical Computing.

[eph13457-bib-0034] Riemann, B. L. , & Lininger, M. R. (2018). Principles of statistics: What the sports medicine professional needs to know. Clinics in Sports Medicine, 37, 375–386. 29903380 10.1016/j.csm.2018.03.004

[eph13457-bib-0035] Roecker, K. , Prettin, S. , & Sorichter, S. (2005). Gas exchange measurements with high temporal resolution: The breath‐by‐breath approach. International Journal of Sports Medicine, 26(1), S11–S18. 15702451 10.1055/s-2004-830506

[eph13457-bib-0036] Rossiter, H. B. (2011). Exercise: Kinetic considerations for gas exchange. Comprehensive Physiology, 1, 203–244. 23737170 10.1002/cphy.c090010

[eph13457-bib-0037] Rossiter, H. B. , Ward, S. A. , Doyle, V. L. , Howe, F. A. , Griffiths, J. R. , & Whipp, B. J. (1999). Inferences from pulmonary O_2_ uptake with respect to intramuscular [phosphocreatine] kinetics during moderate exercise in humans. The Journal of Physiology, 518, 921–932. 10421675 10.1111/j.1469-7793.1999.0921p.x PMC2269465

[eph13457-bib-0038] Sullivan, G. M. , & Feinn, R. (2012). Using effect size‐or why the p value is not enough. Journal of Graduate Medical Education, 4, 279–282. 23997866 10.4300/JGME-D-12-00156.1 PMC3444174

[eph13457-bib-0039] Ward, A. M. M. , Guluzade, N. A. , Kowalchuk, J. M. , & Keir, D. A. (2023). Coupling of V ˙E and V ˙CO_2_ kinetics: Insights from multiple exercise transitions below the estimated lactate threshold. European Journal of Applied Physiology, 123, 509–522. 36371597 10.1007/s00421-022-05073-4

[eph13457-bib-0040] Ward, S. A. (2018). Open‐circuit respirometry: Real‐time, laboratory‐based systems. European Journal of Applied Physiology, 118, 875–898. 29728765 10.1007/s00421-018-3860-9

[eph13457-bib-0041] Whipp, B. J. , Ward, S. A. , Lamarra, N. , Davis, J. A. , & Wasserman, K. (1982). Parameters of ventilatory and gas exchange dynamics during exercise. Journal of Applied Physiology: Respiratory, Environmental and Exercise Physiology, 52, 1506–1513. 6809716 10.1152/jappl.1982.52.6.1506

[eph13457-bib-0042] Wüst, R. C. I. , Aliverti, A. , Capelli, C. , & Kayser, B. (2008). Breath‐by‐breath changes of lung oxygen stores at rest and during exercise in humans. Respiratory Physiology & Neurobiology, 164, 291–299. 18599385 10.1016/j.resp.2008.06.002

